# Prevalence of the human papillomavirus (HPV) types among cervical dysplasia women attending a gynaecological clinic in Sweden

**DOI:** 10.1038/s44276-023-00012-y

**Published:** 2023-08-22

**Authors:** Fabricio Romero García, Johanna Norenhag, Gabriella Edfeldt, Liqin Cheng, Luisa Warchavchik Hugerth, Alexandra A. L. Pennhag, Ina Schuppe-Koistinen, Lars Engstrand, Matts Olovsson, Juan Du

**Affiliations:** 1 Department of Microbiology, Tumor and Cell Biology, Centre for Translational Microbiome Research (CTMR), Karolinska Institutet, Solnavägen 9, 171 77, Stockholm, Sweden; 2 Department of Women’s and Children’s Health, Uppsala University, Sjukhusvägen 7, 753 09, Uppsala, Sweden; 3 Department of Medical Biochemistry and Microbiology, Science for Life Laboratory, Uppsala University, BMC, Husargatan 3, 752 37, Uppsala, Sweden; 4 Science for Life Laboratory, Karolinska Institutet, Tomtebodavägen 23, 171 65, Solna, Sweden

## Abstract

**Background:**

Human papillomavirus (HPV) is the main cause of cervical cancer. HPV-vaccines have led to a significant decrease in HPV-infections and related cancer cases. The estimation of the current HPV-prevalence and distribution of different HPV-types among women with cervical dysplasia is important for the future vaccination strategy.

**Methods:**

By using a multiplexed bead-based immunoassay, we revealed the prevalence of 27 HPV-types in 168 dysplasia women aged 21–70 from Uppsala University hospital, Sweden.

**Results:**

The prevalence of HPV in low-and high-grade squamous intraepithelial lesions (LSIL and HSIL, respectively) were 56.3% and 76.7%, respectively. The oncogenic HPV-types constituted 80.0%, and 97.1% among the HPV-positive LSIL and HSIL-groups, respectively, with HPV16 as the most prevalent type. We found a reduction in oncogenic HPV-types covered by the bi- and quadrivalent vaccines in the vaccinated HSIL-group, suggesting the effectiveness of the HPV-vaccine in preventing dysplasia caused by the covered HPV-types. Oncogenic HPV-types 39 and 59, not covered by any current vaccine have an important prevalence among patients with cervical dysplasia.

**Conclusions:**

Oncogenic-HPV-types are highly prevalent among women with HSIL. The current vaccine presents effectiveness for reducing the covered HPV-types among dysplasia patients.

## Background

According to the International Agency for Research on Cancer (IARC), cervical cancer is one of the most common cancers and a leading cause of cancer death in women [1]. Human papillomavirus (HPV) infection is found in 99.7% of cervical cancer cases worldwide [2]. Studies have shown an HPV-prevalence up to 91.6% in cervical intraepithelial neoplasia (CIN) grade 3 among women in the Nordic countries [3]. HPV-testing has been endorsed as a primary screening method for cervical cancer in Europe [4]. The HPV-vaccine was included in the national vaccination program in Sweden in 2012 targeting 10–12-year-old girls followed by a catch-up vaccination for 13–26-year-old women [5, 6]. Vaccination has been estimated to lead to a reduction in the prevalence of a form of severe dysplasia, cervical intraepithelial neoplasia grade 2 and above (CIN2+), especially for girls vaccinated at a younger age [7]. Moreover, an earlier study with a cohort of young Swedish women between 2006 and 2014 demonstrated that HPV-types covered by the vaccine are rare among the vaccinated individuals regardless of the stage of dysplasia and that the HPV-types not included in the vaccine are associated with CIN lesions [8]. However, surveillance of HPV-types that continuously appear in dysplasia cases is important and needs further investigation.

In the present article, we used a multiplex method to identify the current prevalence of 27 HPV-types among 168 women with histologically verified low-and high-grade squamous intraepithelial lesion (LSIL and HSIL, respectively, the latter including cancer in situ and cancer) 5 to 7 years after introduction of the national HPV-vaccination program in Sweden. In addition, we compared the HPV-prevalence in LSIL and HSIL dysplasia grades with special consideration to age and vaccination status.

## Methods

### Sample collection

In total, 168 women with histological verified cervical dysplasia, according to the Bethesda system, which encompasses moderate and severe dysplasia, namely CIN2 and 3 under the HSIL group [9], were recruited at the gynaecological clinic of Uppsala University hospital, Sweden. Cervical swabs for HPV-testing were collected by physicians during gynaecological examinations between June 2017 and December 2019. Vaccination status was obtained through questionnaires. In total, 82 individuals reported their vaccination status, with 65 patients vaccinated and 17 unvaccinated. All participation was voluntary and pseudonymized, and participants provided the written informed consent. The study was performed according to permissions ethics No. DNR 2016/517 approved by Uppsala Regional Ethics Committee.

### Sample processing

Immediately after sampling, the swabs were preserved in 800 µL DNA/RNA Shield (Zymo Research Corp, CA) and kept at −80 °C. DNA extraction was performed as described previously in our work and stored at –80 °C until HPV-genotyping [10]. DNA/RNA Shield was used as negative control for each extraction procedure. HPV-genotyping was performed on 27 different HPV-types: (1) 15 oncogenic types (HPV16, 18, 31, 33, 35, 39, 45, 51, 52, 56, 58, 59, 68, 73, and 82); (2) six probably oncogenic types (HPV26, 30, 53, 66, 67, and 69); and (3) six non-oncogenic types (HPV6, 11, 42, 43, 44, and 70), following our previous work [10–13]. Briefly, polymerase chain reaction (PCR) amplification with broad-spectrum GP5+/6+ primers targeting the L1 region of the HPV-DNA was employed. In addition, targets for the E6 region of HPV16 and 33 were added to increase the sensitivity and targeted regions. Probes that link to the 27 HPV-types and beta-globin were linked to the PCR products before being submitted to the Luminex^®^ 200^TM^ System (Luminex Inc., TX, USA) as has been performed before in our group [12]. SiHa (HPV16 positive) and HeLa (HPV18 positive) cell DNA extractions were used as positive controls and the water extraction sample as the negative control of the Luminex system [10–13].

### Data analysis

For data analysis, the well values of only water before the PCR run were regarded as background. The raw median fluorescent index (MFI) information was treated using a cut-off of MFI - 15–1.5 × background for all the types except for HPV35, 66, 16, 30, and 18, which were treated with a higher cut-off (MFI - 25–1.5 × background) due to a higher background variation. In total, the data from 168 individuals were used for the analysis. The HPV-risk-groups were defined according to specific characteristics: (1) oncogenic (oHPV), defined as an individual infected with at least one oncogenic HPV-type regardless of any other type(s) the individual was infected with; (2) non-oncogenic (non-oHPV), defined as an individual who was exclusively infected with one or more of the non-oncogenic types; (3) probably oncogenic (pro-oHPV), defined as an individual exclusively infected with one or more of the probably oncogenic HPV-types; or (4) HPV-negative, defined as an individual who was negative for any of the tested HPV-types. Furthermore, women with HSIL were subdivided in Moderate, Moderate/Cancer in situ and Cancer in situ subgroups, according to the grade in the lesion. The mixed subgroup possesses histological characteristics of both moderate and cancer in situ lesions. Among the samples, 82 had information on the vaccination status from which 56 were HPV-positive. The packages tidyverse, ggplot2, waffle, RColorBrewer, and DescTools in R (version 4.2.1, [2022-06-23]) were used to perform the data analysis, the bar-, line-plots and waffle chart, and to perform the G-tests for assessing the statistical significance of the results, respectively.

## Results

### The prevalence of the oncogenic HPV-types increases as with increased severity of dysplasia

**Fig. 1:**
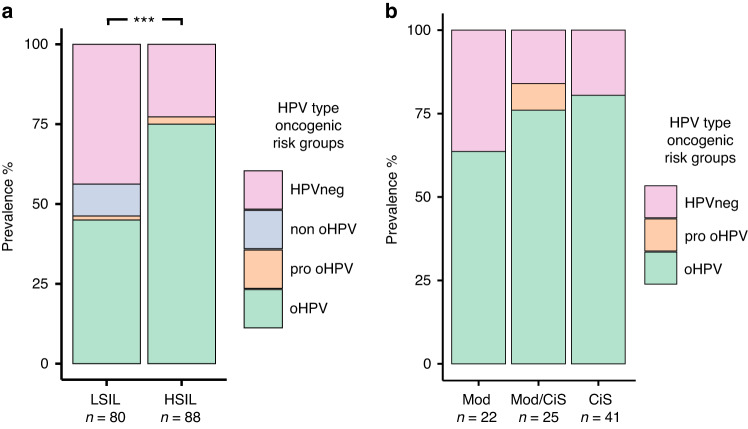
HPV prevalence among different severity of dysplasia. **a** Prevalence of human papillomavirus (HPV) infection grouped by risk among low- and high-grade squamous intraepithelial lesion (LSIL and HSIL, respectively). ***p < 0.01 (based on the comparison on the prevalence of oncogenic HPV types in LSIL individuals with those in HSIL individuals). **b** Prevalence of different HPV types grouped by risk among HSIL subgroups. HPV human papillomavirus, HSIL high-grade squamous intraepithelial lesion, HPVneg HPV negative, non_oHPV non-oncogenic HPV, pro_oHPV probably oncogenic HPV, oHPV oncogenic HPV, Mod Moderate dysplasia, Mod/CiS moderate dysplasia and Cancer in situ, and CiS Cancer in situ.

We first studied the prevalence of different HPV-risk-types among women divided into the LSIL and HSIL dysplasia groups. In total, 45.0% (36 cases) of the women with LSIL (*n* = 80) were infected by oHPV-types and 10.0% (eight cases) by non oHPV-types. Only one woman in the LSIL group had exclusively pro oHPV-types (1.3%), while 43.8% (35 cases) of the women in the group were HPV-negative (Fig. 1a). Notably, the group of women with HSIL (*n* = 88) showed the highest prevalence of the oHPV-types with 75.0% (66 cases) and the absence of non oHPV-types. Furthermore, this group presented two cases with pro-oHPV-types (2.2%), and 20 HPV-negative cases (22.7%) as shown in Fig. 1a. Moreover, when comparing oHPV-prevalence in the LSIL versus the HSIL group, significantly more cases of oHPV in the HSIL than in LSIL group were observed (*p* < 0.01) as shown in Fig. 1a. Further detailed subgrouping based on clinical data of women with HSIL demonstrated that the prevalence of oHPV-types continued to rise corresponding to an increase in the severity of dysplasia. In total, 14, 19, and 33 cases of oHPV-types were found in moderate, moderate/cancer in situ, and cancer in situ subgroups, respectively, constituting 63.6%, 76.0%, and 80.5% of the total cases in the corresponding groups, respectively (Fig. 1b).

### Oncogenic HPV-types are the most prevalent across all ages in women regardless stage of dysplasia

**Fig. 2:**
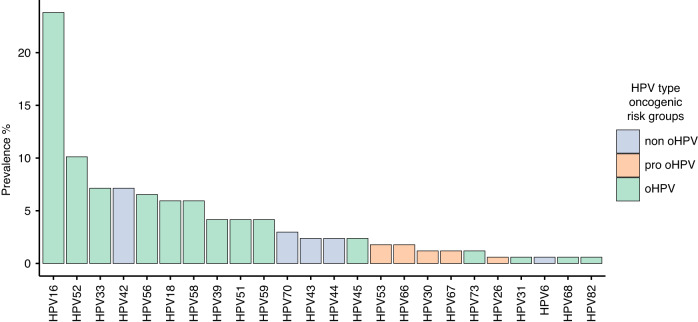
Prevalence of each HPV type and their oncogenic risk among women with dysplasia. Top 24 HPV types are shown and coloured by risk group.

When assessing the prevalence of each HPV-type among the women in the study, we demonstrated that HPV16 was the most prevalent among all the HPV-types (23.8%, *n* = 40) followed by another two oHPV-types HPV52 (10.1%, *n* = 17) and 33 (7.1%, *n* = 12). The most prevalent non-oHPV-type was HPV42, which was found in 7.1% (*n* = 12) of women followed by HPV70 (3.0%, *n* = 5) and HPV43 and 44 (both 2.4%, *n* = 4) as shown in Fig. 2.

**Fig. 3:**
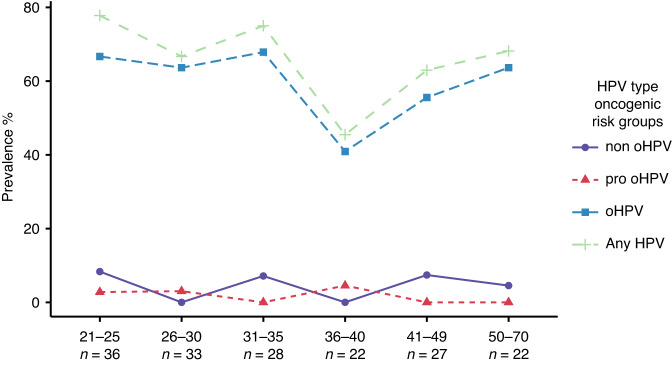
Prevalence of any HPV infection and different HPV risk groups among dysplasia women grouped by age. Nodes represent the prevalence of each age group, and lines depict the trends between the nodes.

We further investigated the prevalence of HPV-infection based on different age groups and found that the overall HPV-infection followed a similar trend as found in the oHPV-infection (Fig. 3). The highest prevalence of any HPV-type was found in the youngest group aged 21–25 years old in which 77.7% (*n* = 28) of the women were infected with at least one type of HPV, and 66.6% (*n* = 24) were infected with one or more oHPV-types. The HPV-prevalence dropped gradually with ageing in individuals and reached the lowest prevalence among individuals aged 36–40 with 45.5% (*n* = 10) in women presenting any HPV-infection and 40.9% (*n* = 9) with at least one oHPV-type. The HPV-prevalence started to increase again after 40 years old and peaked 68.2% (*n* = 15) of any HPV-infection and 63.6% (*n* = 14) of any oHPV-type among women aged 50–70 years old (Fig. 3).

We examined in further detail the distribution of oncogenic HPV-types, covered or not in the vaccines administered, by group of ages with the available information on vaccination status. In total, 47 (72.3%) unvaccinated women and 9 (52.9%) vaccinated women were HPV-positive. Remarkably, the vaccinated individuals of the two youngest age groups, 21–25 and 26–30 years old, did not present oncogenic HPV-infection of the types included in the vaccines, while in unvaccinated women 60.0% (*n* = 6, 21–25 years old) and 33.3% (*n* = 4, 26–30 years old) of individuals were positive for the vaccine-covered types (Table 1). Furthermore, the youngest group of vaccinated individuals showed the highest number of oncogenic-HPV-infection of the types not included in the vaccines (70.0%, *n* = 7) as shown in Table 1.

**Table 1:** Number of cases with HPV types and vaccination status in dysplasia women grouped by age.

Age groups	21–25		26–30		31–35		36–40		41–49		50–70	
Total *n*	*n* = 36		*n* = 33		*n* = 28		*n* = 22		*n* = 27		*n* = 22	
Ind w / vac info	20		17		17		10		9		9	
Status	vac	unvac	vac	unvac	vac	unvac	vac	unvac	vac	unvac	vac	unvac
Total *n* per status	10	10	5	12	2	15	0	10	0	9	0	9
oHPVvac_type	0	6	0	4	1	6	0	2	0	2	0	2
oHPVnon_vac_type	7	2	0	6	0	6	0	1	0	3	0	5
pro_oHPV	0	0	0	0	0	0	0	1	0	0	0	0
non_oHPV	1	0	0	0	0	0	0	0	0	1	0	0
HPVneg	2	2	5	2	1	3	0	6	0	3	0	2

*HPV* human papillomavirus, *Ind w/ vac info* number of individuals for whom vaccination status information is available, *Total n per status* number of individuals per vaccination status, *oHPVvac_type* oncogenic HPV type included in the vaccine, *oHPVnon_vac_type* oncogenic HPV type not included in the vaccine, *pro_oHPV* probably oncogenic HPV type, *non_oHPV* non-oncogenic HPV type, *HPVneg* HPV negative.

### Oncogenic HPV-types among vaccinated women with dysplasia was different compared to the unvaccinated women

**Fig. 4:**
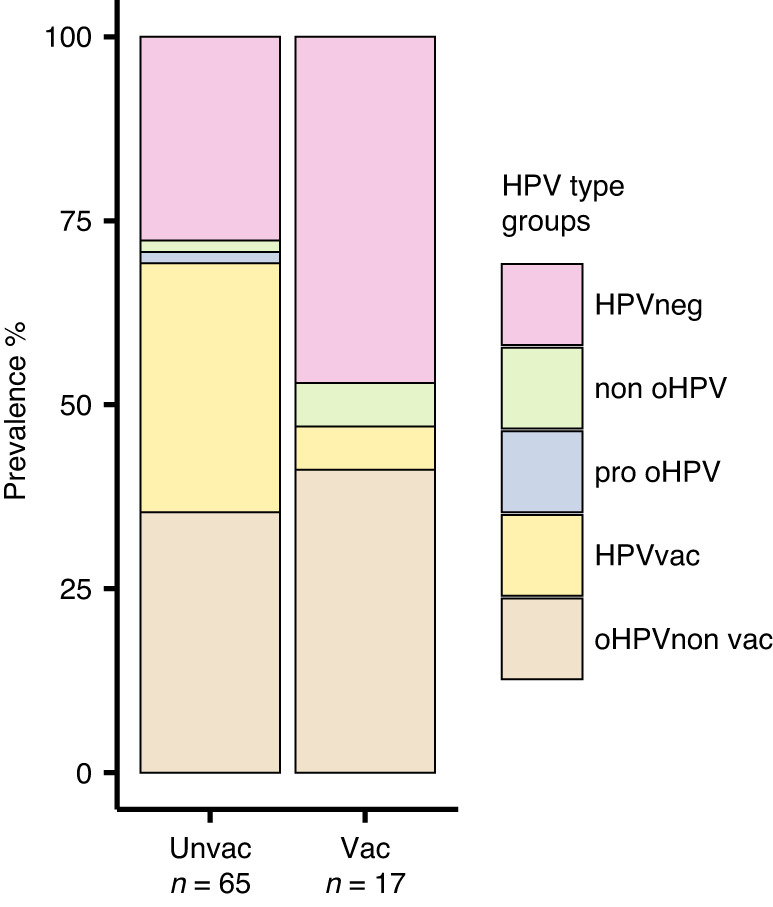
Prevalence of HPV types among vaccinated and unvaccinated women with dysplasia. The barplots display information from 82 individuals who have provided details about their vaccination status. HPVvac HPV vaccine-types, oHPV non vac oncogenic HPV types not covered by the vaccines.

**Fig. 5:**
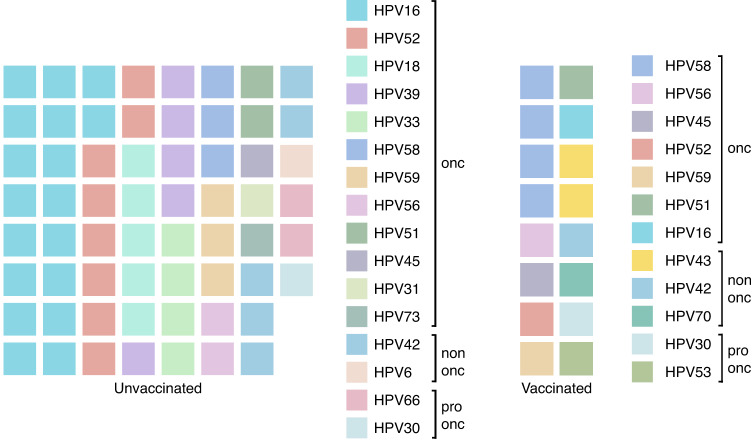
Waffle chart showing the distribution of HPV types in unvaccinated and vaccinated dysplasia women. The number of squares indicates the number of cases per type.

To evaluate further the effectiveness of the HPV-vaccines, we analysed the prevalence of HPV-types covered in the bi- and quadrivalent-vaccine (HPV16, 18, 6 and 11) in the groups with and without vaccination regardless of dysplasia stage. Less women were infected with HPV-types covered in the vaccine among the vaccinated women (*n* = 1) compared to the non-vaccinated women (*n* = 22). The majority of the vaccinated women were positive for the oncogenic-HPV-types not covered in the vaccines administered. Besides, in the group of unvaccinated women, the percentage of individuals infected with HPV-vaccine -covered or -non-covered types were 33.8% (*n* = 22) and 35.4% (*n* = 23), respectively (Fig. 4). Notably, when considering multiple-HPV-type infections per woman, HPV58 (*n* = 4) was the most common oHPV-type in the vaccinated group, while HPV16 (*n* = 18), 52 (*n* = 8), 18 (*n* = 5), and 39 (*n* = 5) were the most prevalent oHPV-types in the unvaccinated group (Fig. 5).

**Fig. 6:**
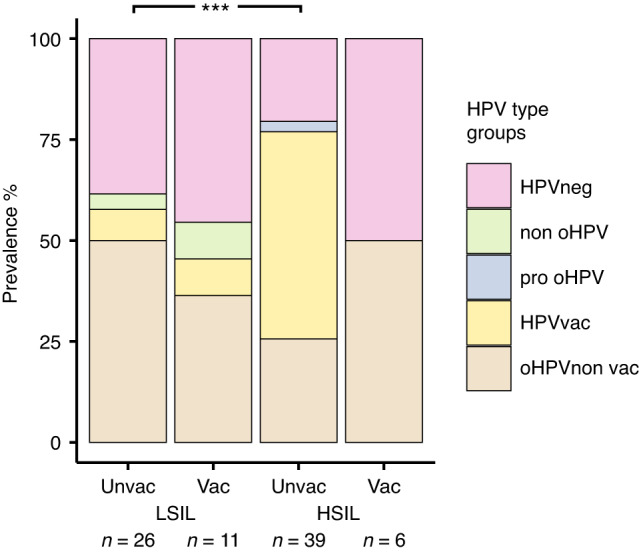
Prevalence of HPV types among vaccinated and unvaccinated women in LSIL and HSIL dysplasia groups. The barplots display information from 82 individuals who provided data on their vaccination status. ***p < 0.01 (based on the comparison on the prevalence of HPV vaccine-types between unvaccinated LSIL individuals and unvaccinated HSIL individuals).

Finally, to obtain a more detailed view of the HPV-types among vaccinated and unvaccinated women with different stages of dysplasia, we further divided both groups into the LSIL and HSIL groups. In the LSIL group, a lower percentage of vaccinated women (36.4%, *n* = 4) compared to unvaccinated women (53.8%, *n* = 14) were infected with oHPV-types not covered in the vaccines administered. However, the situation is different among women with HSIL dysplasia grade, in which 51.3% (*n* = 20) of unvaccinated individuals were infected by one or both of the oHPV-types covered in the vaccines and 25.6% (*n* = 10) by one or more of the oHPV-types not covered. Remarkably, among unvaccinated women, significantly (*p* < 0.01) higher number of individuals infected with oHPV-vaccine-types (HPV16 and 18), were found in the HSIL group in contrast to the LSIL group (Fig. 6).

## Discussion

Our study demonstrated that HPV was detected in over half of the LSIL cases and about three-quarters of the HSIL cases after 5–7 years of the nationwide HPV-vaccination program. Moreover, almost all HPV-positive patients with HSIL possess at least one oncogenic HPV-type. This finding is in-line with previous worldwide meta-analysis, systematic reviews or pooled analysis in which oncogenic-HPV-types are associated with a more advanced stage of dysplasia [14–17]. We also showed that one-quarter of HSIL cases were HPV-negative, which is higher than some of the reports from other countries [18, 19]. This, however, might be due to the differences in vaccination status and/or detection methods. Documented cases of relatively high occurrence of HSIL cases in HPV-negative individuals have also been reported [20]. Nevertheless, the HPV-negative data could arise from histological misclassification, latent or cleared HPV-infection, disruption of the targeting fragment, non-oncogenic HPV-infection, HPV-testing method, or they can constitute cervical adenocarcinoma independent of HPV-infection [21–24].

In our age group analysis, we found the highest HPV-prevalence among the youngest age group of dysplasia patients followed by a steady decline until women reached the age of 40. This finding is in-line to already published papers that report HPV-infection peaks around 20 years old and then it is observed a steady decline along older ages [25]. The second peak after 40 years old might be due to hormonal and immunological changes after menopause, along with potential changes in sexual behaviour, which together, highlights the importance of screening women in this group of age [26, 27].

Furthermore, in Sweden, the quadrivalent-vaccine Gardasil^®^, protecting against HPV-types 16, 18, 6 and 11, was administered in the national program, both school-based and catch-up, while the bivalent-vaccine Cervarix^®^, protecting against HPV-types 16, 18, was additionally available on-demand under the national pharmaceutical products insurance [28]. A considerable number of women in the age groups, up to 33 years, could have received a catch-up HPV-vaccine after the national HPV-vaccination program started. However, they might already have been exposed to HPV-infection before receiving the vaccination. Although, we consider that most of the vaccinated women got the quadrivalent-vaccine, the exact type and age of the vaccination are not available. This situation needs to be considered when interpreting our data as more likelihood of obtaining better protection occurs when the vaccination is given at younger ages [7]. Currently, the HPV-type with the highest prevalence is still HPV16, based on data that are mainly retrieved from unvaccinated patients. However, in vaccinated individuals of our cohort, HPV58, which is only covered in the latest HPV vaccine Gardasil^®^ 9, is the most prevalent. This might be mainly due to Gardasil^®^ 9 not being available during the Swedish HPV-national vaccination program at the time the individuals got their vaccines. Interestingly, we revealed that the youngest group of vaccinated women is free of the oncogenic types included in the quadrivalent-vaccine, while an important number of them were still infected by other oncogenic-HPV-types not included in any of the current three HPV vaccines. This finding is also supported by previous studies in unvaccinated individuals, in which the importance of HPV-vaccination is highlighted [11, 12]. It also underscores the importance of the introduction of vaccines covering more oncogenic types in the Swedish HPV-national vaccination program.

The low prevalence of the oncogenic HPV-vaccine types in vaccinated patients indicate the positive effect of the current vaccine against the covered HPV-types, which is similar with our previous findings from young Swedish women without dysplasia [10]. We also observed an overall decrease in most of the oncogenic-HPV-infections in vaccinated compared to unvaccinated women, and the top HPV-types differ between vaccinated and unvaccinated women. This finding is supported by data from other countries, such as the United Kingdom from which a similar vaccine coverage rate as Sweden was reported and the incidence of HPV-types among women with cervical dysplasia is different compared with pre-vaccinated individuals [6, 29, 30]. In addition, as shown in clinical trials, there might be a cross-protection effect against the oncogenic non-vaccine HPV types 31, 33, and 45 by the quadrivalent-vaccine (versus HPV31) and the bivalent-vaccines (versus HPV31, 33, and 45). This probably explains why we did not observe these types in the vaccinated group [31] (Fig. 5). The fact that the prevalence of HPV-types included in the bi- and quadrivalent-vaccines was higher in HSIL than in LSIL among unvaccinated women points to the role of the oncogenic-HPV-types in the advanced grade of dysplasia and indicates that the current vaccination could potentially reduce more HSIL cases in the future.

Nonetheless, a high prevalence of HPV-infection still exists among the vaccinated women with dysplasia. This prevalence is mainly due to the oncogenic-HPV-types that are not covered in the current vaccines, as we previously reported [10]. In this respect, we found HPV52, 39, and 33 were the top oncogenic-HPV-types present in unvaccinated individuals while HPV58 was the most common oncogenic-HPV-type in the vaccinated women. HPV52, 33, and 58 are already covered in the HPV-vaccine Gardasil^®^-9, which was studied and improved results for preventing LSIL and HSIL [32]. The important prevalence of oncogenic HPV39 and 59, among the dysplasia patients, which are not covered in any current HPV-vaccine, highlights the importance of next-generation vaccine coverage of a greater number of these HPV-types [32].

There are several limitations to our study. Firstly, our sample size is limited, as we only obtained data from patients in one hospital. Additionally, information regarding vaccine types, age, and previous HPV screening results would have been beneficial for interpreting and conducting a more detailed analysis of our data. Another limitation of our study is that the information on vaccination status was gathered through questionnaires, and the response rate for vaccine status was low. A larger cohort studies in multiple hospitals and countries with high HPV-vaccine coverage rates are essential to validate our data.

In summary, our results describe the current HPV-prevalence and distribution of 27 HPV-types among dysplasia women attending a gynaecological clinic in Sweden. We show that the oncogenic-HPV-types are highly prevalent among women with HSIL, and HPV16 is the most common HPV-type among women with dysplasia. The prevalence of oncogenic HPV types increases with the severity of dysplasia and remains high across all age groups in women, regardless of the stage of dysplasia. However, the distribution of oncogenic HPV types among vaccinated women with dysplasia differed from that of unvaccinated women. Considering the different oncogenic HPV types and vaccine status may be useful in tailoring the follow-up time after HPV-screening. Overall, the current vaccines present effectiveness for reducing the covered HPV-types among dysplasia patients.

## Data Availability

The datasets analysed during the current study are available in the Figshare repository, 10.6084/m9.figshare.22731986.v1.
